# Skepticism and excitement when co-designing just-in-time mental health apps with minoritized youth

**DOI:** 10.1016/j.invent.2026.100924

**Published:** 2026-02-21

**Authors:** Caroline A. Figueroa, Kathleen W. Guan, Dimpy Gupta, Neslihan Can, Kayla Green, Jiwon Jung, Eva Thalassinou, Gerben Kuiper, Niko Vegt

**Affiliations:** a Delft University of Technology, Faculty of Technology, Policy, and Management, Jaffalaan 5, 2628 BX, Delft, the Netherlands; b University of California Berkeley, School of Social Welfare, 120 Haviland Hall, Berkeley, CA, 94720, United States; c Delft University of Technology, Faculty of Industrial Design Engineering, Landbergstraat 15, 2628 CE, Delft, the Netherlands; d Erasmus University Rotterdam, Erasmus School of Social and Behavioural Sciences, Burgemeester Oudlaan 50, 3062 PA, Rotterdam, the Netherlands; e Surgery Department, Erasmus MC, University Medical Center Rotterdam, Doctor Molewaterplein 40, 3015 GD, Rotterdam, the Netherlands; f Gro-Up Youth Work, the Netherlands

**Keywords:** Digital mental health, Youth, Adolescent health, Participatory research, Co-design, Just-in-time-adaptive interventions

## Abstract

**Introduction:**

Mental health issues among young people have surged post-COVID-19. Mental health apps can offer accessible preventive support on a large scale, yet the perspective of minoritized youth–such as those from low socioeconomic and ethnic/racial backgrounds–are underexplored. This risks low uptake and effectiveness, and exacerbating health inequities. This study aimed to understand the needs and concerns of minoritized youth in the Netherlands using a participatory approach.

**Methods:**

We conducted 3 co-creation sessions with 17 adolescents (16 females, majority Dutch Moroccan background) aged 11–22 years, recruited through community centers in lower-income neighborhoods in The Netherlands, with the help of community workers. We also organized a discussion session with 26 preventive youth workers to explore their perspectives regarding implementation. A subset of youth (*n* = 10) analyzed the data in 2 co-thematic analysis workshops. We compared youth and researcher themes.

**Results:**

Youth saw data-driven mental health apps as useful for short-term stress relief through motivational quotes, social activity suggestions, and homework support, but unable to solve more severe issues. In the co-analysis, youth analyzed based on emotion and functions, whereas researchers employed a more technical lens. Key themes included identity-based (such as religion, gender, and age) and contextual tailoring (to school/home schedules), compassionate communication as opposed to fake support (robots), safety, and the role of social media.

**Conclusion:**

These findings highlight the need to examine how app design for young people can prioritize authentic, compassionate communication, safety–including transparency about data–tailoring to identify aspects, adapting the timing and frequency of notifications, and integrating social connections and social media. Participatory approaches are promising to better understand the needs of youth from minoritized backgrounds for digital mental health technologies, with the aim of equitable digital solutions.

## Highlights

• Limited knowledge exists on how minoritized adolescents view mental health apps.
• We conducted co-design and co-analysis workshops with minoritized adolescents.
• Youth emphasized compassionate support, identity-based, and social media tailoring.
• Mismatch to schedules, lack of transparency, and distrust of “fake” AI are barriers.
• Findings advocate for culturally responsive, youth-driven digital mental health.

## 1 Introduction

There is a pressing need for new strategies to prevent mental health problems in young people. One in five adolescents (12–25) globally experience mental health problems. Adolescent girls are particularly affected, faring worse than boys across the board in mental health outcomes in Europe, the US and Asia (Cosma, 2023; Rakić et al., n.d.). For instance, more than half of teen girls in the US and Europe feel persistently sad or hopeless (https://hbsc.org/publications/reports/a-focus-on-adolescent-physical-activity-eating-behaviours-weight-status-and-body-image-in-europe-central-asia-and-canada/, n.d.).

Digital mental health interventions (DMHI), particularly smartphone apps, are increasingly developed to address the treatment gap (Figueroa and Aguilera, 2020; Denecke et al., 2022). These apps can offer low-threshold support, including self-management for depression and anxiety, and are scalable among youth–who are avid digital health adopters (Vogels et al., 2022). They increasingly use data-analytics and Artificial Intelligence, such as real-time self-report and sensor data to deliver an intervention when the person needs it most and is most likely to be receptive (e.g., motivating behavior change during heightened risk detection). These so-called Just-In-Time-Adaptive (JITAI) mechanisms could enhance the precision and efficacy of behavior and well-being strategies (Teepe et al., 2021; Guan et al., 2024). By reaching young people on a large scale with personalized and precision support DMHI could make an important contribution to advancing social justice in healthcare (Wies et al., 2021).

### 1.1 Lack of diversity, equity, and inclusion

However, the youth DMHI field struggles with a lack of diversity, equity, and inclusion (DEI). Though mental health apps show promising outcomes, their use and adoption in the real-world is low, especially among minoritized youth, such as those with lower socioeconomic status and ethnic/racial minorities (Bear et al., 2024). For example, only 4% of users continue to use apps after 2 weeks (Baumel et al., 2019). Lack of tailoring to the needs of diverse young people is an important reason (Bear et al., 2024; Stiles-Shields et al., 2023). In most academic studies on DMHI young people are not involved in design processes (Stiles-Shields et al., 2023). Minoritized youth are particularly underrepresented (for example, 71% of youth DMHI research recruited university students) even though they face higher mental health stigma and barriers to care (Figueroa and Aguilera, 2020; Hostetter and Klein, 2022; *Access to Adolescent Mental Health Care, US Office of Populations Affairs Website*, n.d.; Schueller et al., 2019), and experience discrimination both in the online and offline world (Tynes et al., 2020; Tao and Fisher, 2022).

In addition to underrepresentation, DEI considerations, such as language, culturally appropriateness, diverse representation, and tailoring to aspects of identity, which may be more important for minoritzed youth, are often ignored in the design of apps (Ramos et al., 2021). Models to guide research on culturally responsive and equitable mental health apps are lacking (Willis et al., 2022; Borghouts et al., 2021). Taken together, because of these research gaps DMHI may not be as acceptable, feasible and effective for minoritized youth (Borghouts et al., 2021). Knowledge on the needs, wishes, and perceived risks and benefits of emerging mental health technologies for minoritized youth is urgently needed.

### 1.2 Integration of digital mental health with preventive youth services

Further, to promote health equity, the integration of digital mental health interventions with preventive youth services is essential. In the Netherlands, municipalities are responsible for delivering free, preventive child and youth care, which reaches up to 95% of young children through Youth Health Care services ((including services for babies, toddlers, and school-based monitoring) as well as community-based youth and family teams (Vanneste et al., 2022). In addition to these formal services, youth work provides low-threshold, community-based support, often through neighborhood centers, outreach programs, and informal drop-in settings rather than clinical facilities (https://www.hva.nl/binaries/content/assets/subsites/kc-mr/lectoraat-ys/hva---a4-brochure---de-preventieve-kracht-van-het-jongerenwerk-web.pdf, n.d.). Youth workers in the Netherlands support socially vulnerable young people in their personal development and social participation and provide initial mental health support for young people (https://www.hva.nl/binaries/content/assets/subsites/kc-mr/lectoraat-ys/hva---a4-brochure---de-preventieve-kracht-van-het-jongerenwerk-web.pdf, n.d.), including counselling, and referring the young to agencies. However, those from less advantaged families are still not receiving adequate support (Vanneste et al., 2022).Further, approximately 30% of youth care providers experience severe financial difficulties, which puts their existence at risk, and necesstates for them to be innovative, including with digital tools (Blum, 2024; Vos, 2024; Ní Charraighe and Reynolds, 2024). DMHI may enhance the preventive work of youth workers, for example by freeing up their time and supporting early identification (Wong et al., 2020), but the youth workers' perspectives on their use are not well understood (Pawluczuk, n.d.).

### 1.3 Aim

Our research aimed to understand the wishes, needs and concerns regarding mental health apps, with a focus on Just-In-Time-Adaptive Mechanisms, of adolescents with a racial and ethnic minority background and living in lower income neighborhoods in the Netherlands. In addition, we explored the perspective of Dutch youth workers on the value of these apps, as youth workers have an important preventative role in supporting young people at risk for mental health disorders, and referring them to care services (Sonneveld et al., 2022). The youth who were in our study all participated in youth work services. We use a participatory research approach, with a combination of co-design and co-research methods, where youth develop design ideas and co-analyze the data. Our goal of the co-analysis was to compare the existing research codes created by the adult research team with those generated by the youth participants, to find new insights, reflect on the youth's perspective and (in)validate adult researchers' findings. In addition to design ideas, we also provide recommendations for involving minoritized youth in DMHI research.

## 2 Methods

### 2.1 Recruitment

#### 2.1.1 Youths

We recruited youth participants from July to late September 2023, with brainstorming sessions held in September and November 2023, and co-analysis in May–June 2024. We reached out to 15 community centers in the Hague and Rotterdam (major cities in the Netherlands), and two (Dock Rotterdam, and Stichting Jeugdwerk Den Haag) agreed to host sessions with their youth participants. Community youth workers informed the adolescents about the research and invited them to join. Sessions were held at the centers during regular youth activity time slots. Participation was optional, with informed consent (co-signed by parents for those under 16), and each participant received a 20-euro gift voucher per session.

#### 2.1.2 Number of participants per session

**Fig. 1:**
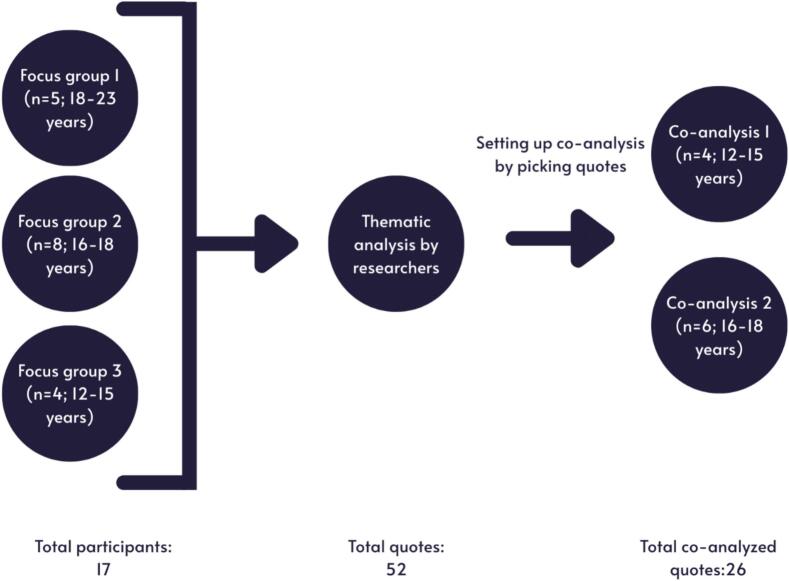
Overview of focus group and co-analysis sessions.

The first session involved five participants aged between 18 and 23 years recruited via Dock in Rotterdam; the second session eight adolescents aged between 16 and 18 years, and the third session four adolescents aged between 12 and 15 years via Stichting Jeugdwerk in Den Haag. We conducted a fourth and fifth session to co-analyze findings from the three focus group sessions; the same youth participated as in session 2 and 3 (4 adolescents 12–16 in the first co-analysis, and 6 (16–18) in the second co-analysis session). An overview of the sessions is shown in Fig. 1.

#### 2.1.3 Ethics

All participants provided written informed consent. For participants under 16 parents additionally provided consent. This research was approved by the Ethical Review Committee of the Delft Technical University.

### 2.2 Methods and materials brainstorm sessions youth

#### 2.2.1 Journey Map, Scenario, and Messages

Before the brainstorming sessions, we qualitatively analyzed 80 randomly selected posts from de Kindertelefoon (kindertelefoon.nl), a Dutch public online forum where adolescents anonymously discuss mental, social, and emotional issues. The posts focused on topics like depression, anxiety, and life events. From this, we created a “journey map” identifying common issues and personas (Carayon et al., 2020). We then developed a persona and scenario based on the journey map, featuring Elin, a 15-year-old girl coping with a stressful home life due to parental conflict, withdrawal from friends, and increased social media use (The supplementary material shows the persona in Dutch and the scenario). The scenario was designed to prompt discussions about coping strategies and mental health app design features. To demonstrate data-driven apps, we illustrated an app that detects user stress and offers real-time techniques to manage stress (such as calling a friend or going for a walk). We also created just-in-time adaptive intervention (JITAI) messages based on prior research (Arévalo Avalos et al., 2024), with additional examples generated by ChatGPT and edited by the researchers.

#### 2.2.2 Co-creation procedure

The co-creation protocol is shown in the supplementary material. We did not use a co-creation framework as directly as the procedure we followed in the co-analysis process, but we can say that we followed the basic mechanisms of co-creation as described by Sanders (2005) (Sanders, 2005). These were led by the design researcher in our team, NV. We began by explaining the study goals, securing informed consent, and distributing vouchers. Participants were shown the scenario of Elin and, in pairs or small groups, asked to reflect on what might help her (initially without mentioning technology). Pens and colored paper were provided for illustrating ideas. Next, we introduced examples of mental health apps using just-in-time adaptive mechanisms to address stress and emotional challenges, asking participants to share their thoughts in groups. We showed a selection of JITAI messages (see supplementary material) and asked them to develop their own app ideas and messages, rating the ones we provided and creating new ones through writing and drawing. The session ended with a reflection on the usefulness of mental health apps, an explanation of how their feedback would inform research and future app development, and a brief overview of available mental health resources. Sessions were conducted in Dutch, audio-recorded and transcribed using Amberscript.ti.

#### 2.2.3 Youth workers

Youth workers filled in a brief questionnaire and provided informed consent. We showed the same scenario (the 15-year-old persona) and asked youth workers to first reflect on what they would do in this situation, second what role technology might play in helping adolescents deal with mental health issues, and third, how apps could support their work. Due to the open nature of these discussion sessions (youth workers could walk in and out of the session, which was held in an open space), we did not audio record this session.

#### 2.2.4 Analysis

We employed an inductive thematic analysis approach following Braun and Clarke's six-step process (Braun and Clarke, 2006): familiarization with the data, generation of initial codes, combining codes into themes, reviewing themes, determining the significance of themes, and reporting the findings (analysis was done in Dutch). This method allowed us to systematically identify and develop emerging themes that encapsulated the needs, wishes, and concerns expressed in the quotes (Naeem et al., 2023). Our approach was fully inductive. We explored how young people themselves described and evaluated mental health apps in general, and our analysis focused on participants' own language and criteria. We utilized Excel and Miro boards for collaborative qualitative coding of anonymous transcripts. The broader research team, which included scientists, medical doctors, and youth workers, engaged in initial discussions to contextualize the findings. Two researchers (DG and NC) first generated codes and themes, which were reviewed and refined in multiple rounds by two other researchers (CAF and NV). DG and NC translated the Dutch codes into English for the purposes of this paper, which was checked by CAF.

The youth worker questionnaire was analyzed descriptively and then compared with observation notes by the researchers of the discussion session with youth workers by CAF and KG.

### 2.3 Co-analysis process

We used a paper on co-analysis with young people by Clark et al. 2022 to guide our co-analysis process (Clark et al., 2022). We chose and printed a proportion of the quotes from the co-design sessions. The selected quotes (*n* = 52) contained young people's ideas on how teenagers could cope with stress, as well as their design ideas for mental health apps with just-in-time adaptive mechanisms (we removed any personally identifying information such as name, location, and age). Additionally, extra information was added in brackets to quotes that were not clear without context. We took pictures of the quotes and codes (see supplementary material).

#### 2.3.1 Co-analysis structure

In line with Clark et al (Clark et al., 2022), we first guided participants through a step-by-step process of the analysis session. We also highlighted the importance of co-analysis and the role of youth within the research. We explained the thematic analysis and a 10–15–minute practice activity to show participants how to group quotes into themes, using the example of pizza topping provided by Clark et al. (Clark et al., 2022). Ideally, we would have conducted this in 2 consecutive sessions, one for training and one for data analysis. Because of time constraints (the youth's schedule with regard to other sessions already planned) we held only one 1,5-h session per youth group.

Next, we divided participants into groups of two or three. Each group received a subset of quotes and had 20 min to code them into themes in several steps, including familiarizing with the data, generating initial codes, determining themes, and forming connections between quotes. After this, we held a 10-minute discussion of the initial youth identified themes. Participants then coded another set of quotes into themes for 20 min before a 10-min wrap-up. In the wrap-up the participants discussed each theme and answered the research question based on their analysis. In the first session, due to an oversight, each group only received approximately half of the quotes. As a result, each group analyzed around half of the quotes (26) instead of the full set.

#### 2.3.2 Analysis

We analyzed the results separately for youth aged 12–16 and youth aged 16–23, and for the co-analysis 12–16 and 16–18, because the older group was not involved in the co-analysis procedure. This decision was based on developmental differences and potential differences in technology use between these groups (Faverio, 2025). It was also based on practical reasons, because the community sessions were already organized according to these age brackets. We used Excel to compare the themes identified by the youth and the researchers. Following the approach described by Clark et al., 4 authors (CAF, NV, DG and NC) held several online meetings to analyze and discuss the codes provided by the youth co-researchers. The codes generated by our youth co-researchers fell into three categories: (1) codes that matched those originally generated; (2) codes that were modified from the original codes; and (3) new codes created by the youth co-researchers (see results section).

#### 2.3.3 Positionality statement

Our personal identities, experiences, and perspectives likely influenced both our methods and analysis. Among the authors, several identify as having a migration background, and one researcher was raised in a Muslim household. Three authors are also young researchers (under 30 years old). One author is a medically trained professional with experience in clinical psychiatry, digital health, and health equity. Four authors are trained designers, with one having extensive experience in participatory research, and one in data-driven design. The brainstorming, discussion, and co-analysis sessions during this study were led by youth with master's studies in industrial design and research supported by senior researchers as background observers. The research team consulted colleagues with expertise in adolescent health, design, and youth work to design the interactive activities and questions posed to youth. All authors are based in the Netherlands, and the majority were raised there. Additionally, two authors have backgrounds as former youth workers. The team consists of cis-gendered hetero men and women. Throughout the research process, we aimed to be mindful of our positionalities and actively engaged in co-analysis to ensure that we did not impose our own interpretations on the data. Our goal was to authentically represent the voices and lived experiences of the youth participants.

## 3 Results

Table 1 shows characteristics of all participants in the study, 17 (mean age = 17.4, SD = 3.0, range = 12–23 years) in total. Most adolescents identified as having a Dutch and Moroccan background (52.9%) and female (94.1%).

**Table 1:** Baseline characteristics of study participants (youth and youth workers).

Youth characteristics	N (%)
Gender identity	
Female	16 (94.1)
Male	1 (5.9)
Age (years)	
Mean age (SD), range	17.4 (3.0), 12–23
10–15	3 (17.6)
16–20	12 (70.6)
20–23	2 (11.8)
Cultural identity	
Dutch and Moroccan	9 (52.9)
Dutch and Turkish	1 (5.9)
Dutch and Arabic	1 (5.9)
Dutch only	2 (11.8)
Turkish only	1 (5.9)
Current education level	
Primary school	1 (5.9)
Secondary school	1 (5.9)
Secondary vocational education	12 (70.6)
Higher professional education	3 (17.6)
Mother's education level	
Secondary school	1 (5.9)
Secondary vocational education	1 (5.9)
Higher professional education	4 (23.5)
Don't know or N/A	11 (64.7)
Father's education level	
Primary school (basis)	1 (5.9)
Secondary school	1 (5.9)
Secondary vocational education	1 (5.9)
Higher professional education	2 (11.8)
University	1 (5.9)
Don't know or N/A	11 (64.7)
Other children in household^a^	
0	4 (23.5)
1	2 (11.8)
2	4 (23.5)
3	4 (23.5)
4	2 (11.8)
N/A	1 (5.9)
Other adults in household^a^	
2	8 (47.1)
3	4 (23.5)
4	4 (23.5)
N/A	1 (5.9)


Youth worker characteristics	N (%)
Age range (years) of youth working with^b^	
<10	9 (39.1)
10–18	16 (69.6)
18–27	11 (47.8)

a Participants answered how many other individuals in their current household (besides themselves) are below (children) or above 18 years (adults) respectively.

b Youth workers provided all age groups they worked with, which may include several age groups.

### 3.1 Themes

Our focus group structure focused on how to deal with stress and difficult situations, and opinions of apps and design ideas. Table 2 shows an example of the codebook of thematic analysis. In total six themes and 40 subthemes were identified.

**Table 2:** Example codebook thematic analysis.

Category	Theme	Subtheme examples	Description	Example quote
Dealing with difficult situations	Human Contact	Support from a psychologist	Positive outlook on talking to a professional/psychologist.	“That she will talk about it with someone who can give her good advice. Someone like a psychologist or someone who is going to give compassionate advice.”
	Dealing with the situation individually	Seeking distraction	Seeking distraction with the help of activities, different environment, or social interactions.	“She could do fun things so that she forgets what's going on at home. For example, going to a community center and playing with friends. Making new friends, new hobbies and, for example, signing up for football if she likes it.”
Opinion on apps and design ideas	App settings	Personalized notifications	The ability to personalize the settings and make sure that the messages are personal.	“Maybe my name will be added. That would feel personal. “Hang in there [name], it's going to be okay.”“
	Other functions	A professional in the app	A feature in the app where the users have access to professional help / the app is linked to professionals, so the app feels more familiar	“If you say things, that he can think of a professional. Not that he's saying you'd better do this or that. Just link it to a professional. That's it.”
	Example notifications	Reminder to connect with a dear one	A message reminding the user to reconnect with a loved one.	“I need a reminder to ask how that one is going. I never do that on my own. [call a friend]”
	Concerns about the app	Privacy concerns	Ensure protection of personal data and privacy with the use of the app such as data breach and data storage.	“Privacy can be very well regulated, but I recently read about a data breach. I don't think anyone would want that. There are all your problems with your name and everything for everyone.”

### 3.2 Dealing with mental health difficulties

Regarding dealing with difficult situations, we identified two main themes: human contact and how to deal with a situation individually.

Youth highlighted the importance of human contact and expressed the importance of talking about their problems with people they trust, like family and friends or school mentors.

““*But her friends can see that something is wrong with her. Then they can ask her: is something wrong? They can give Elin [the persona] some tips and understand her*.”

They also suggested temporarily leaving the home situation:

“*Otherwise, [Elin can] have a conversation with the mentor and explain her home situation. Maybe the mentor can then discuss if she can move on. Then she can go to the library, for example, instead of studying at home*.”

Some youth also stressed the need for the persona Elin to talk to her parents, which many saw as the source of the problem. However, the older youth, stressed that talking to a more distant family member would be more beneficial, such as an aunt or a niece. In addition, professionals like psychologists were encouraged to talk to.

Youth also discussed strategies to deal with mental health struggles individually, through seeking distraction, self-care habits and religious or spiritual practices. For example:

“*That she asks God for help. It depends on what she beliefs*”.

Youth expressed the difficulty of seeking help, and instead withdrawing, mostly with their phones: “ *If I have an argument with my sister or my mother, I also retreat to my room. Then I spend the entire evening on my phone. Then I also isolate myself from the rest*.” Unprompted, the youth did not suggest mental health apps as a solution for mental health problems.

#### 3.2.1 Opinions on apps and design ideas

Researchers identified four broad themes: app settings, additional functions, example notifications, and concerns. App settings covered practical aspects like notification timing, personalization, and JITAI interventions. Other functions included youth-suggested features such as in-app games and links to mental health professionals. For notifications, youth created categories like ‘activating notifications,’ ‘reward-based notifications,’ and ‘reminders to connect with loved ones.’

Many concerns centered on the app's emotional limitations, AI's ability to provide support, and privacy regarding sensitive data. While researchers and youth identified overlapping themes, youth emphasized feelings, user experience, and functionality over technical aspects. They also introduced new themes related to ‘fake support,’ social media, and emotions. Examples of comparisons between researcher and youth-generated themes, along with quotes, is provided in Table 3. The comparison between youth participants and researchers of the entire set of quotes is shown in Supplementary Table 1 and Supplementary Table 2.

**Table 3:** Examples of comparisons between researcher and youth-generated themes.

Theme/subtheme	Example quote	Youth themes 12–16	Comparison to researchers	Youth themes 16–18 years	Comparison to researchers
App settings/ which day messages	“[Times of notifications] On Friday night everyone is happy. You don't need to get happier. On a Wednesday, that's when you know it's not a weekend anymore. You have to get used to school starting again. You have to wash your hair that day, you don't feel like it.”	Ideas for notifications	Modified	Notification reminders	Modified
App settings/ personalized notifications	“You can check the category yourself [in the app]. Lifestyle, health, things like that. From that [the categories] you receive quotes.”	About the app	Matching	Social media Content	New. Apps are discoverable via social media Modified
Concerns about the app/ Short-term effectiveness	“I also think that if you do it digitally [by chatting on an app it] provides stress relief at that time, but it doesn't completely solve the problem.”	Feedback group, solving problems group	Modified	Mindset, psychology Robot	Modified New. This referred to the situation of the persona with fighting parents.
Example notfications/ memories of nice moments	“[An example of an app notification is that] she receives a sweet message, she comes across a reminder and her friends cheering her up.”	Ideas group, non-function group	Matching	Loving	Modified. Youth viewed this as an app cheering you up by reminding you of a nice moment through a message or photo.
Example notifications/religion and identity	“[A quote in the app could be] “Go listen to Allah for one minute plus pink noodles. Don't like pink noodles? Other favourite snack.”“	Ideas for quotes	Matching	Mindset, mental problems	New
Concerns about the app/age limit of an app	“I think that there definitely needs to be an age limit indicated [on the app].”	Ideas group, function group	Modified	Social media Loving Dangers	New New Matching
Concerns about the app/the limitations of bots	Such a bot is indeed not a human being. To what extent is such a bot compassionate?	Feedback group, fake group	New	Mindset, psychology Robot	Modified Matching

#### 3.2.2 Short term effectiveness/feedback to improve apps

Most youth indicated that they are currently not using mental health apps. They expressed that they expect these apps to be useful for short-term mental health support.

*“I also think that if you do it digitally [by chatting on an app], it provides stress relief at that moment, but it doesn't completely solve the problem.”*

This quote was categorized by youth (12–16) as ‘feedback to improve existing apps’, and by the researchers as ‘app effectiveness’.

The short-term effectiveness of apps was also highlighted by the following quote:

“*Yes, I think an app could help her. If she doesn't want to do anything at all, she's on the couch or on her phone. She's on her phone, then she gets a notification: don't you have anything to do? Do this*.”

As feedback for other apps youth also mentioned the accessibility of apps (coded by the researchers as talking to a bot versus a professional):

*“I actually think that [an app] is accessible for young people because the threshold is low. They don't have to immediately have a face-to-face conversation with someone. I think that with such an app, you can go at your own pace.”*

They also highlighted the potential of learning and downloading an app via social media. They coded this as ‘positive feedback’ for apps, which was coded by researchers as ‘discovering an app’.

*“I saw an app via Tiktok, so I liked it, and I downloaded it”.*

#### 3.2.3 Professional referral/information

Referring users to professionals was deemed as a potential app function coded by the researchers with the subtheme ‘professional in the app’, and by youth as ‘mindset’, which they indicated to mean developing a more positive outlook on a situation, either through speaking with a psychologist or through receiving messages that helped them reframe challenges)’: *“[I find] that such an app does allow you to refer to professionals. I would find that an advantage. [...] If you say things, he can think of a professional. Not that he says you better do this or that. Just linking to a professional. That's it.”*

Youth stressed that chatbot apps could be reliable if based on professional information (categorized by youth as ‘mindset’ and by the researcher as ‘bot vs. Professional’:

*“A bot operates on all information, that ultimately comes from articles by scientists and professionals. It's not based on things that are made up. If you put your problem like that and you get advice based on that then it does come from somewhere.”*

Some youth remarked that other teenagers were using AI for chatting about mental health problems, such as MyAI on Snapchat.

**Not a Function:** a new theme emerged, where youth (12–16) expressed that some elements don't belong in a mental health app, such as discussing personal home issues or finding motivation to address mental health challenges.

For example, one participant shared: *“It is important that she can explain what's going on at home and then it's out there. That someone knows she's not feeling well, for example by telling teachers. They take that into account so she can focus on school and classes.”*

Or the difficulty of being motivated to keep using an app: *“..., it is very important that the motivation [to use an app] comes from within. If that is missing, using an app is difficult.”*

### 3.3 Dangers of mental health apps: privacy and age

Youth expressed privacy concerns as an important theme. For example, the researchers' theme *Concerns about the app, privacy concerns,* overlapped with adolescents 16–18 theme *Feedback group, privacy group*. *Participants in the older group (18–23 year years) reflected on the potential use of apps and AI of their younger nieces and nephews.* They highlighted privacy risks and the potential unreliability of AI systems, noting that chatbots can “say anything” without clear boundaries. They discussed the potential need for having an age limit after raising a point about data leaks and privacy issues.

This is illustrated by the following quotes:

*“How does it work with age limits? Is it really appropriate for young people? They can literally say anything.”*

And:

*“When you send a message, where does your message go? Does it stay there? Is it deleted? That's something I value. So, I wouldn't just use an app.”*

A new theme “**Fake”** was devised by the young people (12–16), which the researchers had categorized as an app function (technical). For example, the quote relating to an app that links to professional help: *“If you put a real person behind it, it could be okay. You still wait for that appointment. You use the app to look at the options for now until that time then that's okay. But if you put a bot behind it, no.”*

### 3.4 Youth's design ideas

#### 3.4.1 App features, the role of community and religion

Youth participants suggested several app features, focusing on emotional aspects, while researchers categorized them in a more practical manner. For example, one adolescent said, *“I would not think of a message for myself, but anonymously post a message for other people [on the app].”* Researchers labeled this under “Other app features, community in app,” while youth called it “Feedback group, positive group,” reflecting a difference between user and developer perspectives.

Religion was also important in app features. One participant shared, *“I would personally like that if you download that app, you can indicate if you are Muslim. Then there are all different functions, for example motivation to learn Islamic quotes.”* Researchers coded this as “Example notifications, religion,” while youth labeled it “Ideas group, belief group.”

Both youth groups emphasized that they would only engage with mental health apps if the apps aligned with their identity, including age, gender, and religion.

#### 3.4.2 Mindset

The researchers, like the young people, devised the overarching theme *sample notifications* but devised sub-themes, such as ‘notifications with a reward’:

*“Relax, take a hot shower or bath. Still stressed? Make Camomile tea.”*

This quote was characterized by adolescents 16–18 as a new theme ‘mindset’, a theme they used in relation to mental problems such as stress, difficulty learning, and having nothing to do. The following quote was also classified as ‘mindset’, whereas it was classified by the researchers as app functions/personal notifications:

““*When you have exam week, you have trouble studying or find it very boring. If you can set it up [the app] to receive messages about that. So, you start studying, but at the same time do something fun. Then you feel like studying.”*

#### 3.4.3 Personalization

Youth valued personalization and control about how often and when they receive a notification. For example, youth agreed that notifications should not be sent during school times: *“On weekends it can be around eleven, twelve, so you can still do something with the message the rest of the day, but not during school hours. Work hours are okay”.*

Youth saw the researchers' definition of personalization as feedback on how mental health apps could be improved.

For instance, youth in the 12–16 group devised the theme *Feedback group, positive group*, for the following quote:

*“If I were to use such an app every day for a week and keep getting the same responses, I would think: what's the point?”* was categorized by researchers under the theme of message variation, whereas the youth (12–16) categorized it under the feedback group, problem-solving group.

**Reminders to Seek Connection/loving quotes** ‘Loving’ emerged as a new theme by youth, for example: *“Those messages could work if they suggested activities like going for a walk with friends or reading a book.”,* coded by researchers as ‘reminders to seek connection’.

An interesting quote under the loving theme was, *“I think there should definitely be an age limit indicated [on the app].* Researchers categorized this as a concern, suggesting that youth see setting age limits as a protective, loving gesture.

The loving theme also included actions related to connection, like, *“[An example of an app notification is that] she receives a kind message, sees a reminder, and her friends cheer her up.”*

Examples of specific messages that youth came up to include in an app are shown in Table 4.

**Table 4:** Example notifications/quotes designed by participants.

You are spending too much time on your phone. Go outside and do something fun.
Do you have nothing to do? Go bake a cake or watch a nice movie.
Haven't seen your family in a while? Visit them!
Having trouble studying? Ask your friends for help, or find a quiet place
It's okay not to be okay. Stay strong girl.
Have you asked someone in your surroundings today if he/she is feeling well?
Take a Pickwick Tea and answer the question (on the teabag).
Get your lazy ass up and do something useful. Make yourself useful.
Stay yourself.
Be the person who still tries.
Keep thinking positively.
Be and remain a good person, especially to the ‘bad’ people around you. Be kind-hearted.
Go listen to Allah for 1 min.

### 3.5 Opinions of youth workers on mental health and apps

In the open discussion (not audio recorded) youth workers were skeptical about the efficacy of mental health apps. The majority noted that they do not use them to support their work, nor are they aware of youth using them. Youth workers remarked how while apps can facilitate connections between young people and professionals, provide mental health information and resources, and raise awareness about smartphone usage, they are insufficient on their own. Rather, app functions could help youth find reliable information, which should focus on holistic health (beyond only mental health) and connect them with youth workers, supporting their work. As such, mental health apps may have an important role in not replacing but enhancing and facilitating interactions between youth and youth workers.

These comments from the open discussion were supported by the questionnaire results, where around half of youth workers (*N* = 10, 52.6%) had never recommended digital health resources to youth. Despite low reports of current use, youth workers appeared open to yet uncertain about using mental health apps both in the questionnaire and open discussion. When asked whether mental health apps could support their work, only 33.3% responded “yes”, with most (53.5%) responding “do not know.” Among 14 youth workers who responded to a question about important considerations when developing mental health apps for youth, 85.7% selected relevance to youth's needs 71.4% selected data privacy, and 57.1% selected ease of use. One youth worker additionally warned to prevent youth from “becoming addicted to the internet.”

## 4 Discussion

Overall, youth were both skeptical and excited about the role of apps to support their mental health. In their view, mental health apps could help in the short-term, in momentary stress relief, suggestions for seeking social connections, relaxation, new activities, and practical skills (e.g., homework support). However, these tools are unable to solve more substantial mental health problems. According to the youth in this research, the content of apps should be tailored, with religion and timing, e.g., receiving notifications at the right moment, as important aspects. They saw a role for apps to connect with professionals such as youth workers or psychologists and suggested that apps could be particularly useful in limiting social media time and providing suggestions for offline activities. Our findings highlight that both youth and youth workers see DMHI's potential in momentary support, with a clear warning to not replace human (compassionate) care and support.

Our results are aligned with other research on young people's attitudes to mental health apps and AI, though also provide new perspectives. For example, previous studies found that adolescents have a positive attitude toward and an interest in mental health apps. In a 2024 study roughly 30% of adolescents reported using a mental health app, and over 60% of respondents said they would use one if they had a mental health problem, frequently citing ease of access and availability as key advantages. Other evidence also indicates that adolescents value features such as scientific credibility, security, and user-friendly design when choosing mental health apps (Kabacińska et al., 2022), in line with our findings. Another study among university students who were mental health app users found that while they preferred in-person treatment, users described mental health apps as efficient and helpful, but not as a replacement of in-person care (Holtz et al., 2025), a sentiment shared by the youth participants in our study.

As a unique perspective, youth in this study highlighted the importance of incorporating identity and religion into mental health apps and noted that current apps often lack these elements. They provided innovative ideas such as receiving quotes and games based on religion as a form of support. Though identity is less explored in digital mental health research, other research underscores the need to integrate cultural values, diverse worldviews, and language differences into digital mental health treatments to address mental health issues like depression and trauma (Willis et al., 2022). However, despite evidence showing that religious and spiritual interventions can reduce symptoms of depression and anxiety (Gonçalves et al., 2015), cultural adaptation in mental health apps remains scarce (Bear et al., 2024; Nittas et al., 2024). For example, a 2020 review identified 23 mental health apps for Arabic speakers, only six of which included Islam, and all were rated as low quality (Alhuwail et al., 2020). This underscores a need for frameworks, guidelines and policies on when and how to culturally adapt digital health interventions (Nittas et al., 2024). Some work in co-adaptation (Delfmann et al., 2025) is emerging in this area for other reasearch to build on.

Despite the growing availability of mental health apps, our study shows that both youth and youth workers—who play a key role in preventing mental health disorders—have limited awareness of these tools, and youth workers rarely use them in their practice. This aligns with other research, which highlights that, despite the rapid proliferation of mobile mental health technologies, uptake and engagement among marginalized young people remain low, posing a significant implementation challenge (Bear et al., 2024; Baumel et al., 2019), and many youth are unaware of the existence of mental health apps (Høgsdal et al., 2024).

As far as we are aware, no other studies have examined youth perspective on data driven just in time adaptive interventions. Overall, young people found just-in-time adaptive interventions (JITAIs) acceptable–particularly in ensuring that messages are delivered at optimal times of the day, such as in the morning and after school– but were more critical about digital tools involving AI. Both young people and youth workers emphasize that these systems should have clear boundaries and functions, such as referring young people to professionals.

Regarding conversational AI, studies have found that young people have positive perceptions of digital conversational agents (Digital Conversational Agents for the Mental Health of Treatment-Seeking Youth: Scoping Review). Other research indicates that young people rely on mental health support from consumer-focused generative AI chatbots, such as ChatGPT, and report these tools are helpful (Siddals et al., 2024; McBain et al., 2025). In our study, young people provided a new perspective in the co-analysis, which showed that support from apps can be deemed caring, but also inauthentic, for example when mental health care is provided by automated systems (robots). There is a need for further research to evaluate the benefits and limitations of conversational AI, such as user experience studies to explore hybrid models combining AI and human interaction for mental health support, especially as these technologies are advancing rapidly (Siddals et al., 2024).

The skepticism among both youth and youth workers also calls for a need for a deeper understanding of the unique barriers these groups face in accessing and using mental health apps, as well as the types of services young people prefer—whether traditional or digital. Since there currently is insufficient evidence on the effectiveness of digital mental health for minoritized youth (Bear et al., 2024), this must be followed by more rigorous and consistent demonstrations of the feasibility, effectiveness, and cost-effectiveness of these digital interventions (Bear et al., 2024).

Our findings, combined with previous research, highlight the need to examine how app design can consider emotional safety, including transparency about data, providing caring support, identity and religion, and integrating human connection. As far as we know, we are the first to conduct a co-analysis process to examine young people's perspective of mental health apps. The youth's categorization process was more user-centered and focused on emotion, with new themes like ‘fake support” (a dislike of robots), ‘loving and peaceful’ (ensuring apps are safe and providing human connection), compared to the more technical and functional approach of the researchers. Our analysis also showed that youth participants crave information on how apps handle their data, desire a minimum age requirement for the app, and see social media as a way to discover a new apps. The difference we find between youth and adult researchers shows the importance of participatory methodologies and design processes to ensure that findings of researchers adequately reflect the youth perspective. This is in line with calls from other research to better incorporate co-design and participatory methods (Chinsen et al., 2025), which also make participating in research more enjoyable and meaningful for young people (Delfmann et al., 2025; Bülow et al., 2025).

### 4.1 Recommendations for involving minoritized youth in digital mental health research

Based on our experiences, we offer suggestions for including minoritized youth in research, who are often deemed ‘hard to reach’. We made a conscious effort to reach underrepresented young people, and included mainly Moroccan-Dutch adolescent girls, who face higher and different types of mental health problems than white Dutch youth (Stomp, 2023).

First, the recruitment strategy played an essential role. Rather than relying on conventional methods such as social media advertisements or university-based recruitment, we purposefully collaborated with community-based organizations. Identifying organizations willing to participate was time intensive but offered advantages. For instance, adolescents already knew the other group members, leading to a level of comfort in the group, and did not need to travel for the sessions, increasing the likelihood of young people participating in research. We were able to reach a group likely less familiar with the topic than young people reacting to an advertisement, providing a new perspective. The presence of a youth worker, who participated and at times gave the youth additional explanations, facilitated the discussions.

Second, the research timeline required adaptation to meet the diverse needs and schedules of the youth participants. Especially the youth over 16 had competing priorities, such as school exams. Further, our research partly took place during the Ramadan, which delayed the sessions. While extending the timeline would allow for greater flexibility, funding constraints make this approach challenging. In research planning, researchers should consider this need for flexibility.

Third, in reflections after the sessions, youth stressed the importance of relationship building throughout the process, and suggested researchers should engage in activities outside of the research. Some of the youth found the co-analysis process to remind them too much of school activities, highlighting the importance of developing playful and interactive methods of conducting digital health research with teenagers. Some resources for this are already available, and include interactive storytelling tools, maker toolkits (Anders and Stappers, 2012) and prototyping techniques (van der Velden et al., 2016). Other studies have also emphasized the importance of youth-led approaches and revealed similar challenges, such as the time commitment required to build relationships, funding limitations, and an academic culture that values individualism as opposed to participatory approaches (Clark et al., 2022; Delfmann et al., 2025). Future research should prioritize addressing barriers to youth engagement and developing principled approaches for conducting meaningful co-research with young people, particularly in the context of digital mental health.

### 4.2 Limitations

Involving young people in thematic analysis revealed both challenges and unique insights. A key limitation was that youth had limited time to analyze and categorize the quotes compared to the researchers. Reducing the volume of quotes in future sessions, or allowing more time for the analysis process, could alleviate this issue and enable a more in-depth analysis. Additionally, youth's living circumstances, the role of language, digital and health literacy, or potential disabilities were not part of our co-creation sessions, all of which are critical factors for digital health engagement (Bear et al., 2024). This should be incorporated into future research. Further, adolescent boys were underrepresented in this study, which limits the generalizability of findings. Moreover, participants were already engaged in weekly group sessions on mental health and personal development, which may have predisposed them to greater interest and familiarity with the topic compared to broader adolescent populations.

Finally, this research focused on adolescents' ideas and needs through design research rather than examining their real-world interaction with existing mental health apps. Their expressed preferences may not align with their actual app usage behaviors. More research on real-life engagement with digital mental health tools might uncover other factors of importance for digital mental health design for minoritized young people.

### 4.3 Conclusion

Through a process of participatory research, we provide valuable information on the understanding of the potential and limitations of data-driven mental health apps for youth (mainly girls) with ethnic minority backgrounds living in lower income neighborhoods in the Netherlands. Overall, mental health apps were seen by both young people and preventive youth workers as a short-term form of support. For young people this support can be deemed loving and caring, but also inauthentic, especially when mental health care is automated (robots) without a human perspective. Digital mental health interventions may be able to cater to the needs of diverse youth by fitting with their identity (e.g. age, gender, religion), social media experiences, and their daily context through just-in-time mechanisms. Mental health apps will likely have limited effects on the mental health of minoritized youth if they are not designed according to their wishes, experiences, and context. More research on the perspective of minoritized youth on emerging health technologies is necessary, followed by research to demonstrate the effectiveness of these technologies in currently underrepresented groups.

## Declaration of Generative AI and AI-assisted technologies in the writing process

During the preparation of this work the author(s) used chatGPT in order to check the readability of paragraphs. After using this tool/service, the author(s) reviewed and edited the content as needed and take(s) full responsibility for the content of the published article.

## Funding

This project was funded by a Convergence Healthy Start Sprint Grant. CAF and KG are supported through the ‘High Tech for a Sustainable Future’ capacity building programme of the 4TU Federation in the Netherlands. The funders had no role in the content of the manuscript.

## Declaration of competing interest

The authors declare the following financial interests/personal relationships which may be considered as potential competing interests: Caroline Figueroa reports financial support was provided by Convergence Healthy Start. Caroline Figueroa and Kathleen Guan report financial support was provided by 4TU Federation in the Netherlands. If there are other authors, they declare that they have no known competing financial interests or personal relationships that could have appeared to influence the work reported in this paper.

## Data Availability

Data will not be made publicly available as it concerns data of adolescent participants.

## Appendix A Supplementary data

Supplementary material Image 1

## References

[bb0005] Access to Adolescent Mental Health Care, US Office of Populations Affairs Website. https://opa.hhs.gov/adolescent-health/mental-health-adolescents/access-adolescent-mental-health-care.

[bb0010] Alhuwail D., Albaj R., Ahmad F., Aldakheel K. (2020). The state of mental digi-therapeutics: a systematic assessment of depression and anxiety apps available for Arabic speakers. Int. J. Med. Inform..

[bb0015] Anders E., Stappers P.J. (2012).

[bb0020] Arévalo Avalos M.R. (2024). The effect of cognitive behavioral therapy text messages on mood: a micro-randomized trial. PLOS Digit. Health.

[bb0025] Baumel A., Muench F., Edan S., Kane J.M. (2019). Objective user engagement with mental health apps: systematic search and panel-based usage analysis. J. Med. Internet Res..

[bb0030] Bear H.A. (2024). The acceptability, engagement, and feasibility of mental health apps for marginalized and underserved young people: systematic review and qualitative study. J. Med. Internet Res..

[bb0035] Blum K. (2024). https://healthjournalism.org/blog/2024/09/what-to-know-about-fdas-new-digital-health-advisory-committee/.

[bb0040] Borghouts J. (2021). Barriers to and facilitators of user engagement with digital mental health interventions: systematic review. J. Med. Internet Res..

[bb0045] Braun V., Clarke V. (2006). Using thematic analysis in psychology. Qual. Res. Psychol..

[bb0050] Bülow A. (2025). From burden to enjoyment: a user-centered approach to engage adolescents in intensive longitudinal research. J. Adolesc..

[bb0055] Carayon P., Wooldridge A., Hoonakker P., Hundt A.S., Kelly M.M. (2020). SEIPS 3.0: human-centered design of the patient journey for patient safety. Appl. Ergon..

[bb0060] Chinsen A. (2025). Co-design methodologies to develop mental health interventions with young people: a systematic review. Lancet Child Adolesc. Health.

[bb0065] Clark A.T., Ahmed I., Metzger S., Walker E., Wylie R. (2022). Moving from co-design to co-research: engaging youth participation in guided qualitative inquiry. Int J Qual Methods.

[bb0070] Cosma A. (2023).

[bb0075] Delfmann L.R. (2025). Experiences with a co-creation process to adapt a healthy sleep intervention with adolescents: a health CASCADE process evaluation. Public Health.

[bb0080] Denecke K., Schmid N., Nüssli S. (2022). Implementation of cognitive behavioral therapy in e-mental health apps: literature review. J. Med. Internet Res..

[bb0085] Faverio M. (2025). https://www.pewresearch.org/internet/2025/12/09/teens-social-media-and-ai-chatbots-2025/.

[bb0090] Figueroa C.A., Aguilera A. (2020). The need for a mental health technology revolution in the COVID-19 pandemic. Front. Psychol..

[bb0095] Gonçalves J.P.B., Lucchetti G., Menezes P.R., Vallada H. (2015). Religious and spiritual interventions in mental health care: a systematic review and meta-analysis of randomized controlled clinical trials. Psychol. Med..

[bb0100] Guan K.W. (2024). Just-in-time adaptive interventions for adolescent and young adult health and well-being: protocol for a systematic review. BMJ Open.

[bb0105] Høgsdal H., Kyrrestad H., Rye M., Kaiser S. (2024). Exploring adolescents’ attitudes toward mental health apps: concurrent mixed methods study. JMIR Form. Res..

[bb0110] Holtz B.E., Kanthawala S., Martin K., Nelson V., Parrott S. (2025). Young adults’ adoption and use of mental health apps: efficient, effective, but no replacement for in-person care. J. Am. Coll. Heal..

[bb0115] Hostetter M., Klein S. (2022).

[bb0120] A focus on adolescent physical activity, eating behaviours, weight status and body image in Europe, central Asia and Canada. https://hbsc.org/publications/reports/a-focus-on-adolescent-physical-activity-eating-behaviours-weight-status-and-body-image-in-europe-central-asia-and-canada/.

[bb0125] https://www.hva.nl/binaries/content/assets/subsites/kc-mr/lectoraat-ys/hva---a4-brochure---de-preventieve-kracht-van-het-jongerenwerk-web.pdf.

[bb0130] Kabacińska K. (2022). What criteria are young people using to select mobile mental health applications? A nominal group study. Digit. Health.

[bb0135] McBain R.K. (2025). Use of generative AI for mental health advice among US adolescents and young adults. JAMA Netw. Open.

[bb0140] Naeem M., Ozuem W., Howell K., Ranfagni S. (2023). A step-by-step process of thematic analysis to develop a conceptual model in qualitative research. Int J Qual Methods.

[bb0145] Ní Charraighe A., Reynolds A. (2024). Re-thinking youth work as initial mental health support for young people. Child. Soc..

[bb0150] Nittas V., Daniore P., Chavez S.J., Wray T.B. (2024). Challenges in implementing cultural adaptations of digital health interventions. Commun. Med..

[bb0155] Pawluczuk A. Automating Youth Work: Youth Workers Views on AI. https://pjp-eu.coe.int/documents/42128013/116591216/AI_views+of+youth+workers.pdf/93ac326a-cf80-3fa4-c4e5-56ee4038a766?t=1682336763487.

[bb0160] Rakić, J. G. *et al.* A focus on adolescent physical activity, eating behaviours, weight status and body image in Europe, central Asia and Canada. Health Behaviour in School-aged Children International Report From the 2021/2022 Survey. 4.

[bb0165] Ramos G., Ponting C., Labao J.P., Sobowale K. (2021). Considerations of diversity, equity, and inclusion in mental health apps: a scoping review of evaluation frameworks. Behav. Res. Ther..

[bb0170] Sanders E.B.N. (2005). Proceedings of the 6th International Conference of the European Academy of Design.

[bb0175] Schueller S.M., Hunter J.F., Figueroa C., Aguilera A. (2019). Use of digital mental health for marginalized and underserved populations. Curr. Treat. Options Psychiatry.

[bb0180] Siddals S., Torous J., Coxon A. (2024). ‘It happened to be the perfect thing’: experiences of generative AI chatbots for mental health. Npj Ment. Health Res..

[bb0185] Sonneveld J., Metz J., Manders W., Schalk R., Van Regenmortel T. (2022). The contribution of professional youth work to the personal development and social participation of socially vulnerable youngsters: a Dutch longitudinal cohort study. Child Adolesc. Soc. Work J..

[bb0190] Stiles-Shields C., Ramos G., Ortega A., Psihogios A.M. (2023). Increasing digital mental health reach and uptake via youth partnerships. NPJ Ment. Health Res..

[bb0195] Stomp D.O. (2023). https://www.verwey-jonker.nl/artikel/discriminatie-schaadt-mentale-gezondheid-van-jongeren-met-migratieachtergrond/.

[bb0200] Tao X., Fisher C.B. (2022). Exposure to social media racial discrimination and mental health among adolescents of color. J. Youth Adolesc..

[bb0205] Teepe G.W. (2021). Just-in-time adaptive mechanisms of popular mobile apps for individuals with depression: systematic app search and literature review. J. Med. Internet Res..

[bb0210] Tynes B.M. (2020). Trajectories of online racial discrimination and psychological functioning among African American and Latino adolescents. Child Dev..

[bb0215] Vanneste Y.T.M., Lanting C.I., Detmar S.B. (2022). The preventive child and Youth Healthcare service in the Netherlands: the state of the art and challenges ahead. Int. J. Environ. Res. Public Health.

[bb0220] van der Velden M., Sommervold M.M., Culén A., Nakstad B. (2016). Perspectives on HCI Research with Teenagers.

[bb0225] Vogels E.A., Gelles-Watnick R., Massarat N. (2022).

[bb0230] Vos S. (2024). https://www.binnenlandsbestuur.nl/sociaal/rapport-stand-van-de-jeugdzorg-2024-schetst-weer-somber-beeld.

[bb0235] Wies B., Landers C., Ienca M. (2021). Digital mental health for young people: a scoping review of ethical promises and challenges. Front. Digital Health.

[bb0240] Willis H.A. (2022). Culturally responsive telepsychology & mHealth interventions for racial-ethnic minoritized youth: research gaps and future directions. J. Clin. Child Adolesc. Psychol..

[bb0245] Wong C.A. (2020). Digital health technology to enhance adolescent and young adult clinical preventive services: affordances and challenges. J. Adolesc. Health.

