# Supplementary material for: Skepticism and excitement when co-designing just-in-time mental health apps with minoritized youth
Source: Internet Interv. 2026 Feb 21;43:100924. doi: 10.1016/j.invent.2026.100924 (PMC12955585; doi:10.1016/j.invent.2026.100924)
Supplement: Supplementary file 1 — Supplementary material [file mmc1.docx]

Supplementary material belonging to Figueroa et al.

1. **Example quotes shown to youth to facilitate the discussion:**

I*t seems that you spend a lot of time on social media and feel overwhelmed. Maybe you can do something offline.*

*It seems loud in your environment and that you are stressed. Maybe wearing headphones will help?*

*It seems like there is tension at home. A fun family game might help.*

*It looks like you haven't seen your friends in a while. Plan a movie night.*

*It seems that you missed school. Ask a friend what you missed.*

1. **Persona and scenario’s:**

*Persona in Dutch (persona was later translated to ‘Elif’ per suggestion of the participants).*

*
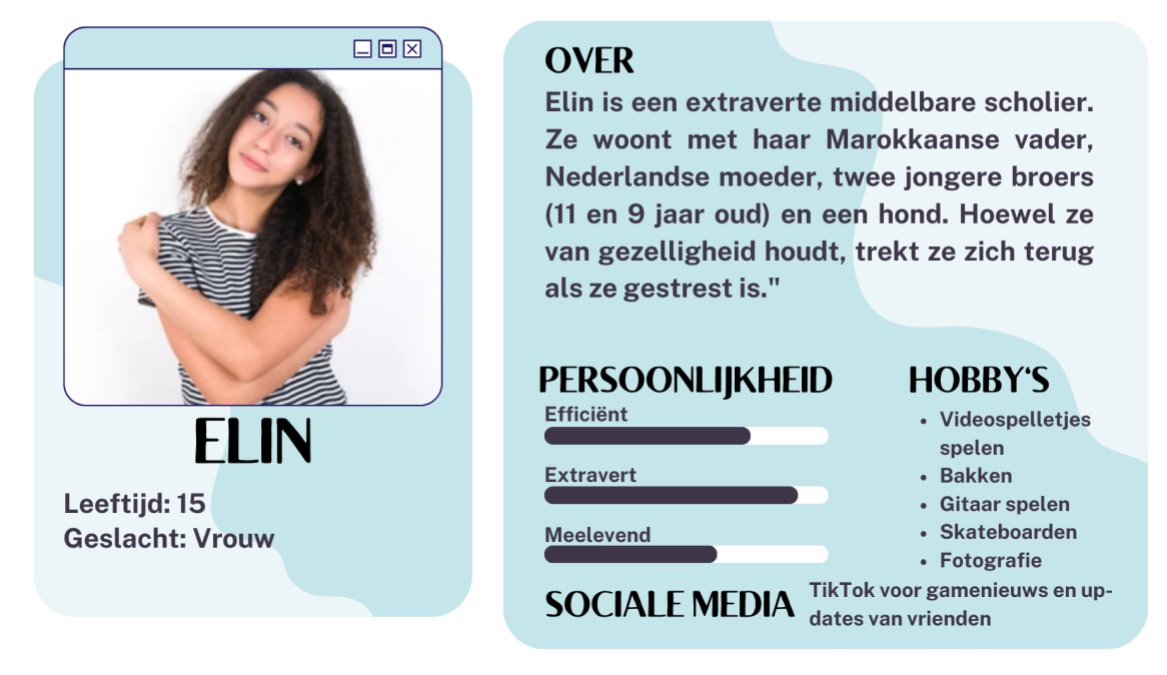
*

 Visual Scenario’s:
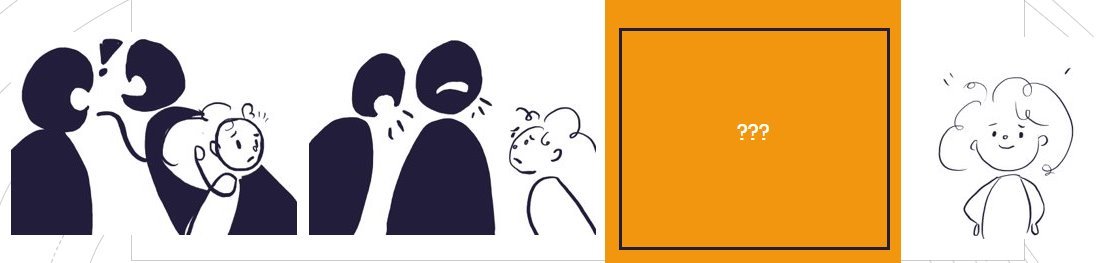

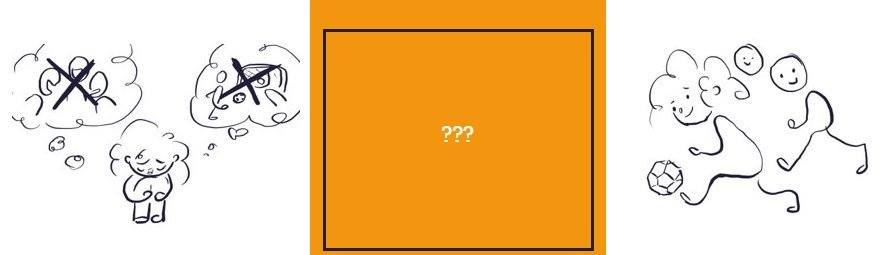


*Participants were asked to reflect on what the persona Elin could do to feeling better and becoming motivated to conduct activities she enjoyed before.*

***
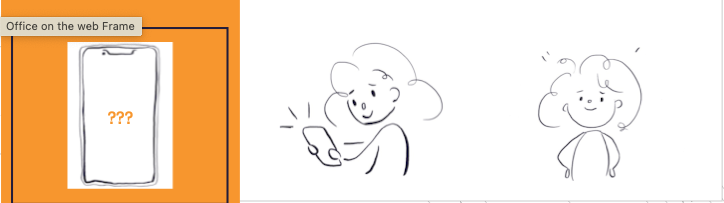
***

*Participants were asked to reflect on what the persona Elin could do on her phone to make her feel better.*

***3. Co-creation Protocol***

**Part 0: INTRODUCTION (15 min)**

- Consent form
- Demographics survey
- Post-its
- Introduction of what we do with examples + Ice breaker
- Hand out vouchers

**Part 1 (25 min)**

- Explanation Persona Elin (5 min)
- Explanation scenario without the mental health app
- Is everything clear?

Brainstorm in groups of thee: Original scenario scene, blank space, revised scenario scene (10 min)

- Arguing parents, blank space, relieved/destressed Elin
- Grades getting lower, blank space, motivated Elin
- Elin not going out with friends/playing sports, blank space, Elin with friends and doing sports

How Brainstorm? Participants must fill in the blanks:

- Provide paper with coloured pencils and drawings for blank spaces
- Divide participants in groups of 3
- Ask: What could have happened that made Elin happy/rested/etc.?

Presentation (participants), ask questions (interviewers) (10 min, 3 min p/group, 1 min pitch, 2 min questions?)

- Present your scenarios, which one do you think is the best one?
- Why do you think this is the best scenario, compared to your other scenarios?
- Why does this make Elin feel better?

**Part 2 (30min)**

- Explanation scenario with mental health app but without just in time (2min)
- Elin receives something on her phone, which makes her look like the 3 possible revised Elin's again. What could she have done on her phone?
- Fill in the blanks: how to fill in the app. (5min)
- Create messages which would help Elin to reach this scenario
- Explain just –in-time mechanic: show examples (2min)
- Sorting 5 messages
- Now, make up messages which would help Elin (5-10)
- Present both scenarios (10-15min)
- What do you think about this?
- Would it be useful for Elin?
- Do you think Elin would use this just once or longer? What would help her make it more sustainable?
- How do you like the just-in-time vs your own creation?

**Part 3 (10 min)**

- Final conclusion, summary and our vision, what next?
- Thanks + any questions?
- Reflection: did your perspective change in regards of using a phone/messages to help someone in need?
- If yes, when did this happen?
- Why did this not happen? Can you give arguments for your opinion?
- Mental health resources
- Contact: Mail

**Supplementary Table 1. Hierarchy of Themes Identified by Researchers and Youth Participants**

| Researcher Theme / Subtheme | Youth Themes (12-16 years) | Youth Themes (16-18 years) |
| --- | --- | --- |
| Other app features (Game, Community, Professional, Examples of other apps) | Ideas for new apps, faith, Feedback, (giving feedback to others); fake (robots are fake); function group (this could be an app function), positive feedback for apps (what could make apps better) | Mindset (developing a more positive outlook), mental problems; Dangers; psychology |
| App settings (Timing, Just-in-time, Notifications, Screen time) | Ideas for app notifications; About the app; Ideas for quotes for the app; About yourself (working on your own goals) | Problems; Content; Screentime; Notification reminders; Social media |
| Feelings after notification | Ideas and quotes for the app | Mindset, psychology; Dangers; Notification reminders |
| How to discover an app | Positive feedback for apps | Notification reminders; Via TikTok |
| Dealing with the situation individually | About yourself (working on your own goals) | Mindset (developing a more positive outlook), mental problems; Screentime |
| Short-term effectiveness | Feedback group, solving problems (inability of apps to solve all mental health problems) | Robot; Mindset, psychology |
| Human contact (Core issues, Psychologist, Confidant) | Talking in real life; Ideas for the app, non-function group (this should not be an app function) | Mindset, psychology; Open book, honesty; Peace; Don’t agree |
| Personal interpretation of messages | Ideas group, non-function group | Open book, honesty; Mindset, mental problems |
| Variety in messages | Feedback for the app, problems solving group; Ideas group, function group | Mindset, mental problems; Critical |
| Example notifications (Memories, Rewards, Connection, Religion) | Ideas group, non-function group; quotes for the app; function group; About yourself; Ideas group, religion | Loving; Peace; Mindset, mental problems; Content; Social media; Notification reminders |
| Concerns about the app (Digital behavior, Age limit, Bots, Potential harm, Privacy) | Ideas group, non-function group; function group; Feedback group, fake group; privacy group; solving problems group | Mindset, mental problems; Dangers; Loving; Robot; Content; psychology |
| Short term effectiveness (Bot vs. Professional) | Feedback group, positive group; About the app; Talking in real life | Via TikTok; Mindset, psychology |

**Supplementary Table 2. Full comparison of researcher and youth themes.**

| Theme/subtheme | Example quote | Youth themes 12-16 | Comparison to researchers | Youth themes 16-18 years | Comparison to researchers |
| --- | --- | --- | --- | --- | --- |
| Other app features, game in the app | “Or that you get quizzes about your faith, for example. That you get all the questions and can click on a, b, c, for example. That you can play that with a family member.” | Ideas group, faith group | Modified | Content |  |
| Other app features, community in the app | ‘’I would not come up with a message for myself, but anonymously post a message for other people [on the app]. That people post messages there.’’ | Feedback group (giving feedback to others through an app), positive group | Modified | Not taking any action  Mindset, mental problems  Dangers | New  New    New. Youth remarked that many are critical of using an app for mental health. |
| Other app features, a professional in the app | ‘’If you put a real person behind it, it might still be possible. That you still wait for that appointment. That you use the app to look at the possibilities for now until then, then that's okay. But if you put a bot behind it, no.’’ | Feedback group, fake group | New | Dangers | New |
| Other app features, a professional in the app | "[I find] that such an app does allow you to refer to professionals. I would find that an advantage. [...] If you say things, that he can think of a professional. Not that he says you better do this or that. Just linking to a professional. That's it." | Ideas group, function group | Matching | Mindset, psychology | Matching. Here youth remarked it’s better to talk to a psychologist [than use an app] to work on your goals. |
| Other app features, examples of other apps | ‘’That was another one of those apps where you could put your emotion under each day of the week. At the end of the week, you got a recap of all your feelings that week. That was kind of a nice idea.’’ | Feedback group, positive group | Modified | Content |  |
| App settings, which day messages | ‘’[Times of notifications] On Friday night everyone is happy. You don't need to get happier, then you don't need it. On a Wednesday, that's when you know it's not a weekend anymore. You have to get used to school starting again. You have to wash your hair that day, you don't feel like it.’’ | Ideas for notifications | Modified | Notification reminders | Modified |
| App settings, feedback on personal notifications | ‘’ If you have test week, you have trouble learning or you find it very boring. If you can set up that you get messages from that. That you are going to learn, but then do something fun at the same time. That you feel like learning.’’ | - | - | Mindset, mental problems | New |
| App settings, feedback on personal notifications | ‘’Here [in a notification in the app] they say: you did that and it's like that. While you yourself can think: no, that's not right.’’ | Ideas for notifications | Matching | Mindset, psychology | New |
| App settings, just in time | ‘’ Just at certain times [send a notification], like when you've really done your best. Then you're really going to feel it, but not if someone says it to you every day.’’ | Ideas for notifications | Modified | Notification reminders | Matching |
| App settings, just in time | ‘’ Or you go to those [mental health] apps yourself and look at questions when you really have nothing to do.’’ | About the app | Modified | Mindset, mental problems | New |
| App settings, personalized notifications | ‘’ Maybe my name will be included [in the app's quote]. That would feel personal. ‘Hang in there [name], it will be fine.’’’ | Ideas group, quotes for the app group | Modified | Content | Modified |
| App settings, personalized notifications | ‘’ You can check the category yourself [in the app]. Live style, health, things like that. So from that you get phrases.’’ | About the app | Matching | Social media  Content | New. Apps are discoverable via social media  Modified |
| App settings, screen time | ‘’ That you stop doing your daily work because you are too focused on that app. Maybe a screen time could be installed in that app. That you can spend a few minutes or an hour a day on it.’’ | About yourself | New | Mindset, psychology | New. This is about working on your own goals |
| App settings, time of messages | ‘’On weekends, [the app can send you a notification] around eleven, twelve o'clock, so you can still do something with the message the rest of the day, but not during school hours. Work hours can be.’’ | Ideas for notifications | Modified | Screentime  Notification reminders | Matching  Matching |
| Feelings after notification | ‘’Sometimes it does contain positive things. For example, "Love yourself." Things like that. That's kind of nice to see. Suppose you had a fight in the morning and you're very angry. Then you go on your phone and you see that. Then there's a smile on your face again anyway. For a moment, not for long.’’ | Ideas group, quotes for the app group | Modified | Content | New |
| Feelings after notification | ‘’ It is programmed in such a way that every day you get to read a sentence they want you to read. To make you feel kind of good. If I am angry and read that [keep your peace], I think: get lost. What should I do with this?’’ | Ideas group, quotes for the app group | Modified | Mindset, psychology  Dangers | Modified  Modified |
| Feelings after notification | [A notification from the app] is yet another check: keep your peace. You don't want to stress. Stressing is not good for you in so many ways | Ideas group, quotes for the app group | Modified | Notification reminders,  Dangers | Modified  New |
| How to discover an app | ‘’ I saw it [an app] on TikTok and liked it, so I went and downloaded it.’’ | Feedback group, positive group | Modified | Social media  Notification reminders  Via tiktok | Modified  New  Modified |
| Dealing with the situation individually, difficulty taking action on one's own | ‘’ It's hard to take action yourself, though, I think [when things are bad for you].’’ | About yourself | Matching | Not taking action  Mindset, mental problems | Matching  Modified |
| Dealing with the situation individually, withdrawing with mobile | ‘’If I don't feel the need to have a conversation during the breaks, I also isolate myself. Then I'm also on my phone. That's really my time and I sometimes like that.’’ | - |  | Screentime | New |
| Short-term effectiveness | ‘’I also think that if you do it digitally [by chatting on an app it] provides stress relief at that time, but it doesn't completely solve the problem.’’ | Feedback group, solving problems group | Modified | Mindset, psychology  Robot | Modified  New. This referred to the situation of the persona with fighting parents. |
| Human contact, discussing the core | ‘’ I myself would vent right away. I am a person who is not going to do that over the app or over the phone. I tell the person directly that he has hurt me. This is what I'm dealing with.’’ | Talking in real life | Matching | Open book, honesty  Mindset, psychology | New  Matching |
| Human contact, psychologist support | ‘’ That she is going to talk about it with someone who can give her good advice about it. Someone like a psychologist or someone who will give compassionate advice.’’ | Talking in real life | Matching | Peace  Mindset, psychology | New  Matching |
| Human contact, personal boundaries in support | ‘’ That doesn't seem so nice, talking to someone you don't know.’’ | Talking in real life | Matching | Don’t agree | Modified |
| Human contact, confidant | ‘’ That she can explain what is happening at home and then it is out. That someone knows she is not feeling well, telling teachers, for example. That they take that into account so she can focus better at school and during classes.’’ | Ideas group, non-function group | New | - | - |
| Personal interpretation of messages | ‘’ I think you can be critical yourself in what advice you take or don't take.’’ | Ideas group, non-function group | New | Open book, honesty  Mindset, mental problems  Critical | New  Modified    Modified |
| Variety in messages | ‘’ If I were to go on such an app every day for a week and repeat the same things over and over again, I would continuously get the same answers. I would then think: what should I do with this?’’ | Feedback group, problems solving group | Modified | Mindset, mental problems  Critical | New    New |
| Variety in messages | ‘’ A variation of both, not that you get assignment every day. That you also get reminders.’’ | Ideas group, function group | Matching | Content | Matching |
| Example notfications, memories of nice moments | ‘’ [An example of an app notification is that] she receives a sweet message, she comes across a reminder and her friends cheering her up.’’ | Ideas group, non-function group | Matching | Loving | Modified. Youth viewed this as an app cheering you up by reminding you of a nice moment through a message or foto |
| Example notifications, notifications with a reward | ‘’ [A quote from the app could be] ''are you hungry? Make your favorite dish. Are you sitting on the couch or on your phone with nothing to do? Find a nice movie, make snacks and go watch.”’’ | Ideas group, quotes for the app group | Matching | Loving | New |
| Example notifications, notifications with a reward | ‘’ [A quote in the app could be] "Relax, take a hot shower or bath. Still stressed? Make camille tea."’’ | Ideas for quotes | Matching | Peace  Mindset, mental problems | New  New |
| Example notifications, notifications with a reward | [A quote from the app could be] Or you deserve dessert today or something . | Ideas for quotes | Matching | Mindset, mental problems | New |
| Example notifications, reminder to connect | ‘’ Those notifications might work if it says, for example, that you should go for a walk with your friends today or read a book.’’ | Idea group, function group | Matching | Loving | New |
| Example notifications, reminder to connect | ‘’ [A quote from the app could be] ''Haven't seen your family in a while? Visit them and bring them a game.”’’ | Ideas for quotes | Matching | Content | Matching |
| Example notifications, reminder to connect | ‘’ [about calling a friend] I need a reminder to ask how she's doing. I never do that on my own.’’ | About yourself | New | Content | Matching |
| Example notifications, activating notifications | ‘’ I have an app for when I'm on my phone too much and doing nothing. Then they say, for example: go outside and get some fresh air. I already have that app and I like it, but I also like this app.’’ | Ideas for quotes | Matching | Do not agree | New (opinion) |
| Example notifications, activating notifications | ‘’ Sometimes I'm on TikTok and I see quotes and that motivates me so much.’’ | Ideas for quotes | Matching | Social media  Notification reminders  Via tiktok | New  Modified  New |
| Example notifications, empowering notifications | ‘’ [A quote that worked for me was] that you should never become a bad person because of the reasons that made you feel bad. You shouldn't change because of people and what they have done. I found that something that really worked for me.’’ | Ideas for quotes | Matching | Mindset, mental problems | Modified |
| Example notifications, religion/identity | ‘’ I would like it if you download that app, so that you can indicate whether you are Muslim. Then there are all different bars, for example motivation to learn, Islamic, quotes.’’ | Ideas group, religion group | Matching | Content | Modified |
| Example notifications, religion/identity | ‘’[A quote in the app could be] "Go listen to Allah for one minute plus pink noodles. Don't like pink noodles? Other favourite snack."’’ | Ideas for quotes | Matching | Mindset, mental problems | New |
| Concerns about the app, digital behaviour | ‘’ Motivation is something very important in this. You have intrinsic and extrinsic motivation. The moment someone goes to a healthcare provider there is already extrinsic motivation. The moment you want to use an app, it is very important that the motivation comes from within yourself. If that is missing, using such an app is difficult.’’ | Ideas group, non-function group | New | Mindset, mental problems  Dangers | Modified    Matching |
| Concerns about the app, age limit of an app | ‘’ I think that there definitely needs to be an age limit indicated [on the app].’’ | Ideas group, function group | Modified | Social media  Loving  Dangers | New  New  Matching |
| Concerns about the app, the human limitations bot | ‘’Such a bot is indeed not a human being. To what extent is such a bot compassionate?’’ | Feedback group, fake group | New | Mindset, psychology  Robot | Modified  Matching |
| Concerns about the app, the human limitations bot | ‘’ [Calling] is much more personal. You also connect faster. You actually hear a voice with intonation. You don't have that here [app].’’ | Idea group, function group | New | Content | New |
| Concerns about the app, potential harm of the app | ‘’ If an app is not developed properly, I think it can even cause damage. To what extent can professional artificial intelligence be used?’’ | Feedback group,  Fake group | New | Mindset, mental problems  Dangers | Modified    Matching |
| Concerns about the app, potential harm of the app | ‘’ Excessive use can also become a problem, I think. If someone feels very comfortable with such an app and starts using it too much, making it no good.’’ | Feedback group, solving problems group |  | Mindset, mental problems | Modified |
| Short term effectiveness, talking to a bot vs a professional | ‘’ I actually think it [an app] is accessible to young people because the threshold is low. They don't have to go face to face and talk to someone right away. I think with an app like that, you can go at your own pace.’’ | Feedback group, positive group | Modified | Mindset, psychology  Via tiktok | Matching  New |
| Short term effectiveness, talking to a bot vs a professional | ‘’ Usually they are accumulations of. The moment you want to talk to such a bot, nine times out of ten you immediately tell them what is bothering you at that moment. Usually it goes much deeper than that. I think a psychologist can deal with that better. A psychologist works with you towards a goal.’’ | Talking in real life | Matching | Mindset, psychology | Matching |
| Short term effectiveness, talking to a bot vs a professional | ‘’ A bot does operate on all information. This is information that ultimately comes from articles by scientists and professionals. It is not stuff that is made up. If you put your problem like this and you get advice based on that, it does come from somewhere.’’ | About the app | Modified | Mindset, psychology  Via tiktok | Matching  New |
| Concerns about the app, privacy concerns | ‘’ I wouldn't share my feelings online. By accepting cookies, they already know so much about us. Then I'm on the phone with someone about glasses and a few minutes later I see those glasses appear on TikTok. I'm not into that myself.’’ | Feedback group, privacy group | Matching | Mindset, psychology  Robot | New  New |
| Concerns about the app, privacy concerns | ‘’ Privacy can be very well regulated, but I read recently about a data breach. I don't think anyone would want that. Then there are all your problems with your name and everything in front of everyone.’’ | Feedback group, privacy group | Matching | Content | New |
| Concerns about the app, privacy concerns | ‘’ Where is it going? When you send a message, where does your message go? Will it stay there? Will it be removed? That is something I do value. So I wouldn't just use an app.’’ | Feedback group, privacy group | Matching | Mindset, psycholgy | New |
